# Reciprocal natural hybridization between *Lycoris aurea* and *Lycoris radiata* (Amaryllidaceae) identified by morphological, karyotypic and chloroplast genomic data

**DOI:** 10.1186/s12870-023-04681-2

**Published:** 2024-01-02

**Authors:** Miaohua Quan, Xianghui Jiang, Longqian Xiao, Jianglin Li, Juan Liang, Guanghua Liu

**Affiliations:** 1 College of Biological and Food Engineering, Huaihua University, Huaihua, 418008 China; 2 Key Laboratory of Hunan Province for Study and Utilization of Ethnic Medicinal Plant Resources, Huaihua University, Huaihua, 418008 China; 3 Key Laboratory of Hunan Higher Education for Hunan Western-Medicinal Plant and Ethnobotany for Western Hunan Medicinal Plant and Ethnobotany, Huaihua University, Huaihua, 418008 China

**Keywords:** *Lycoris*, Natural hybrid, Karyotype, Chloroplast genome, Evolution of species

## Abstract

**Background:**

Hybridization is considered as an important model of speciation, but the evolutionary process of natural hybridization is still poorly characterized in *Lycoris*. To reveal the phylogenetic relationship of two new putative natural hybrids in *Lycoris*, morphological, karyotypic and chloroplast genomic data of four *Lycoris* species were analyzed in this study.

**Results:**

Two putative natural hybrids (2n = 18 = 4 m + 5t + 6st + 3 T) possessed obvious heterozygosity features of *L. radiata* (2n = 22 = 10t + 12st) and *L. aurea* (2n = 14 = 8 m + 6 T) in morphology (e.g. leaf shape and flower color), karyotype (e.g. chromosome numbers, CPD/DAPI bands, 45S rDNA-FISH signals etc.) and chloroplast genomes. Among four *Lycoris* species, the composition and structure features of chloroplast genomes between *L. radiata* and the putative natural hybrid 1 (*L. hunanensis*), while *L. aurea* and the hybrid 2, were completely the same or highly similar, respectively. However, the features of the cp genomes between *L. radiata* and the hybrid 2, while *L. aurea* and the hybrid 1, including IR-LSC/SSC boundaries, SSRs, SNPs, and SNVs etc., were significantly different, respectively. Combining the karyotypes and cp genomes analysis, we affirmed that the natural hybrid 1 originated from the natural hybridization of *L. radiata* (♀) × *L. aurea* (*♂*), while the natural hybrid 2 from the hybridization of *L. radiata* (*♂*) × *L. aurea* (♀).

**Conclusion:**

The strong evidences for natural hybridization between *L. radiata* (2n = 22) and *L. aurea* (2n = 14) were found based on morphological, karyotypic and chloroplast genomic data. Their reciprocal hybridization gave rise to two new taxa (2n = 18) of *Lycoris*. This study revealed the origin of two new species of *Lycoris* and strongly supported the role of natural hybridization that facilitated lineage diversification in this genus.

**Supplementary Information:**

The online version contains supplementary material available at 10.1186/s12870-023-04681-2.

## Background

*Lycoris* Herb. (Amaryllidaceae) is a herbaceous perennial plant. The genus consists of more than twenty species and mainly distributes in eastern Asia, particularly in China, among which *L. aurea* and *L. radiata* are the most widespread species [1–3]. Its bulb is rich in alkaloids, which is of important medicinal value such as tumor-suppressing and anti-malarial etc. [4]. At the same time, flowers of this genus are diverse in shape and colour such as pink, red, white, yellow and multicolor etc., it has been used as an ornamental plant for centuries [5].

*Lycoris* species are considered to have high genetic diversity [3]. To date, a satisfactory consensus classification for *Lycoris* remains elusive due to frequent hybridization and wide morphological variation [5]. In the past years, interspecific relationships, karyotype and hybridization of *Lycoris* species have been widely performed [6–9]. The basic chromosome number of the genus was *n* = 6, 7, 8 or 11, with the ploidy ranging from euploid (diploid, triploid, tetraploid) to aneuploid [7, 10], e.g. *Lycoris aurea* possessing 12, 13, 14, 15, 16, 18 chromosomes with karyotypes of median centromeric point (M-type), median region (m-type), terminal point (T-type), terminal region (t-type) and subterminal region (st-type) etc. [11–13]. *Lycoris radiata* has great variation in karyotype and chromosome numbers throughout its geographical range, including diploid (2n = 22), triploid (2n = 33), tetraploid (2n = 44) and aneuploid (2n = 21,32) etc. [9]. The chloroplast (cp) genome basically follows maternal inheritance and is commonly used to reveal the maternal parent of hybrids [14, 15]. Some species of *Lycoris* have been recently confirmed to be of hybrid origin by the cp genome and karyotype etc. For example, *L. haywardii* originated from the interspecific hybridization of *L. sprengeri* and *L. radiata* var. *Pumila* [11, 16]. *L. flavescens* may be originated from a hybridization of *L. sanguinea* and *L. chinensis* [17]. At the same time, some new hybrid taxa were successively reported, such as *L. houdyshelii* and *L. hubeiensis* etc. [7, 18]. However, phylogenetic relationship of *Lycoris* has been an open question due to frequent hybridization and continuous variation of morphological and physiological characteristics in this genus [8, 10, 19]. This evolutionary process of natural hybridization is still poorly characterized in *Lycoris*.

During field investigations of germplasm resources of *Lycoris* in Hunan and Hubei Province etc., China*,* we found two new natural variant populations of *Lycoris* with cream or chalky yellow flower in the distribution area of *L. radiata* and *L. aurea*, which have never been reported in these regions, and speculated that the two variations might represent undescribed taxa based on the observation of flower shape and colour. In this study, the living bulbs of the two new variant populations, *L. radiata* and *L. aurea* distributed in the same region (Yuanling County) were collected and studied on the karyotypes by CPD staining (combined PI and DAPI), fluorescence in situ hybridization (FISH) and chloroplast genomes by high-throughput sequencing etc. in order to reveal the interspecific relationships of the four *Lycoris* species, which could provide a scientific basis for the phylogenetic relationships and karyotype evolution of *Lycoris*.

## Results

### Morphology comparison

**Fig. 1:**
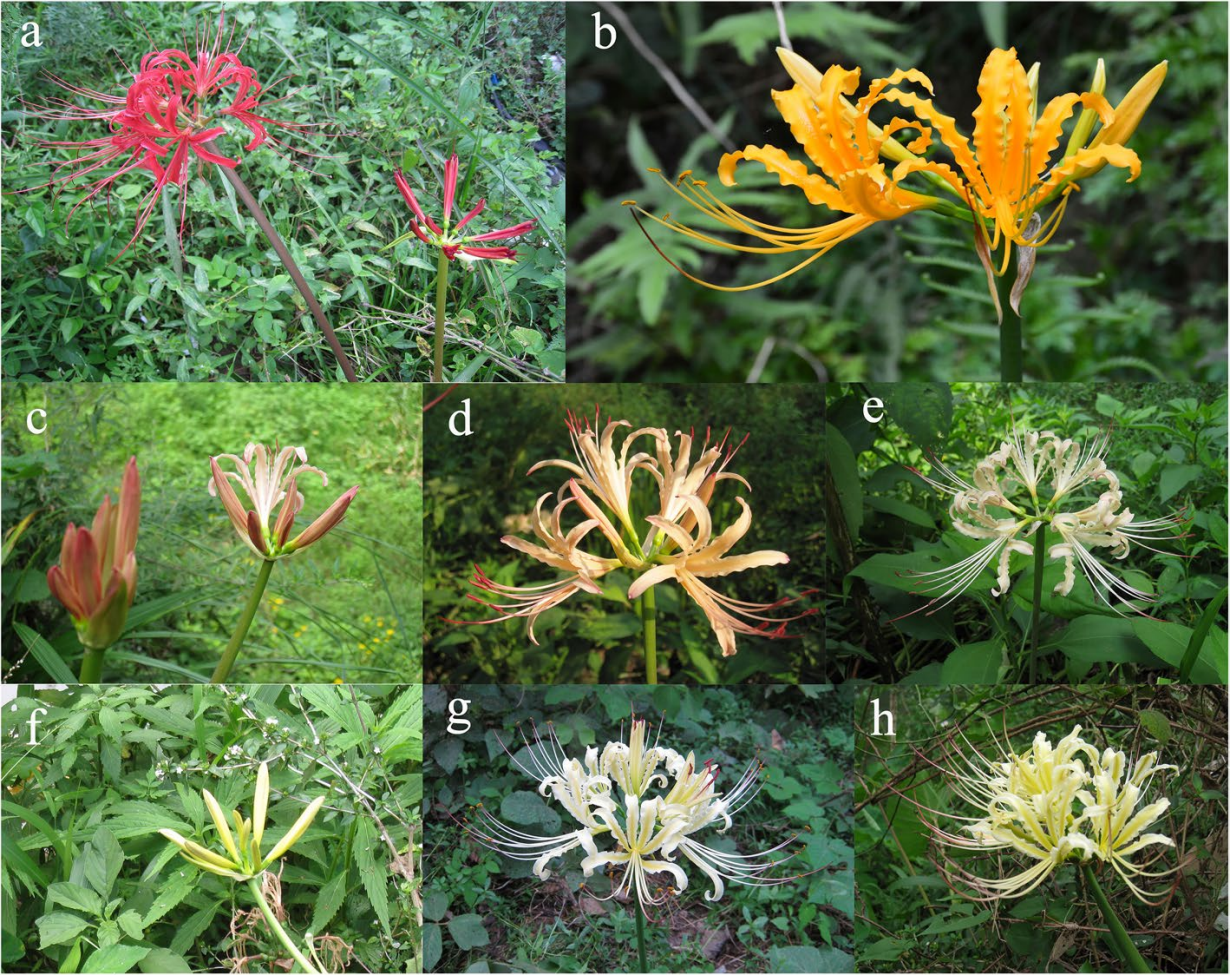
The flowers of four taxa of *Lycoris* investigated in this study. **a** *Lycoris radiata*; **b** *Lycoris aurea*; **c**-**e** putative natural hybrid 1 (*L. hunanensis*), including bud (**c**), flower in early anthesis (**d**) and in mid-anthesis (**e**); **f**–**h** putative natural hybrid 2, including bud (**f**), flower in early anthesis (**g**) and in mid-anthesis (**h**)

The mainly morphological characteristics of *L. radiata* (Fig. 1a), *L. aurea* (Fig. 1b), putative natural hybrid 1 (Fig. 1c-e) and putative natural hybrid 2 (Fig. 1f-h) were shown in Fig. 1 and Table 1. As shown in Table 1, leaf widths of *L. radiata, L. aurea,* natural hybrid 1 (*L. hunanensis*) and hybrid 2 were 0.9, 3.2, 1.5, and 1.8 cm respectively. The flower colors of the hybrid 1 and hybrid 2 were gradually changing from pink or pale yellow in bud to cream or chalky yellow with few scattered red stripes in mid-anthesis respectively, and also the mature fruits and seeds have not been observed etc., suggesting that the two new natural variant taxa might be hybrid generations of *L. radiata* and *L. aurea*.

**Table 1:** Morphological characteristics of the four samples in *Lycoris*

Species	Leaf			Flower color			Abilities of developing mature fruit
	Length (cm)	Width (cm)	Apex	Bud	Early anthesis	Mid-anthesis	
*L. radiata*	45.8 ± 5.3	0.9 ± 0.2	obtuse	red	red	red	can
*L. aurea*	55.6 ± 7.5	3.2 ± 0.4	acuminate	yellow	yellow	yellow	can
Natural hybrid 1	48.5 ± 6.2	1.5 ± 0.2	acuminate	pink	lightly yellowish pink	cream, with scattered red stripes	Cannot^a^
Natural hybrid 2	52.3 ± 5.4	1.8 ± 0.3	acuminate	pale yellow	light yellow	chalky yellow, with scattered red stripes	cannot

^**a**^Fruit and seed of the putative natural hybrid 1 (*L. hunanensis*) have not been observed during cultivation. The traits of fruit and seed described in *L. hunanensis* [20] were inaccurate, which were made correction and amended in this study. Five plants (three leaves each plant) each sample were selected to determine morphological traits

### Karyotype analysis

**Fig. 2:**
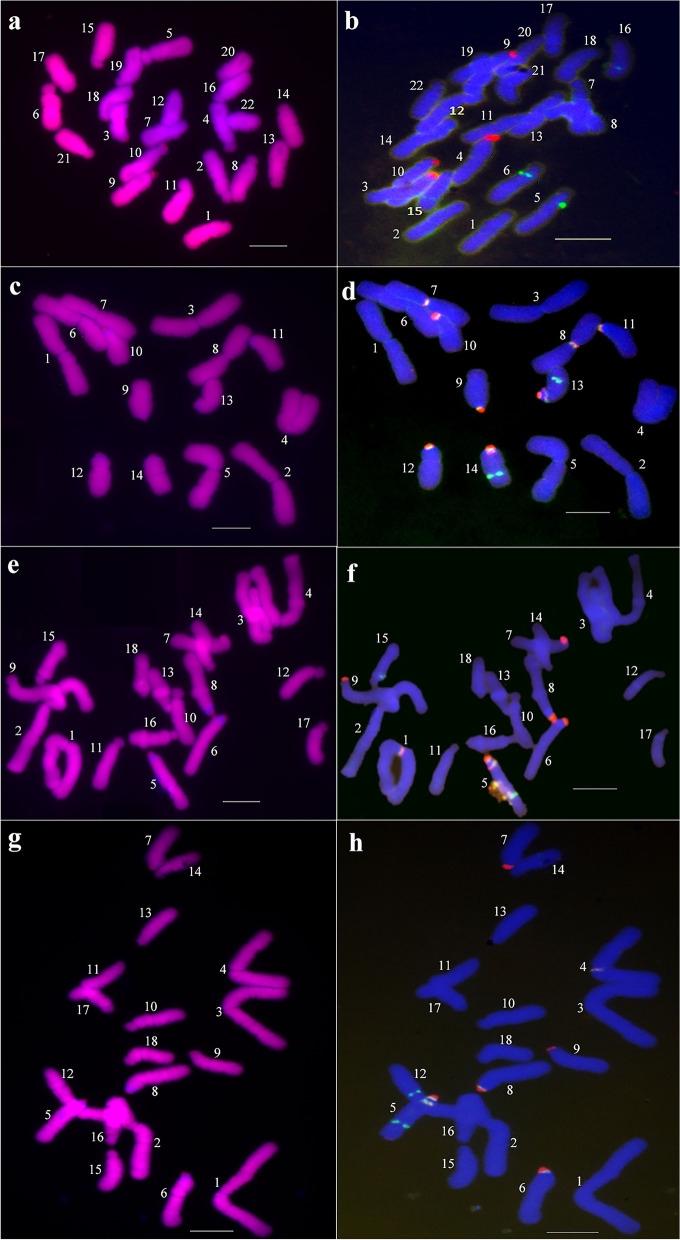
Chromosomes of four *Lycoris* species stained with CPD and 45S rDNA-FISH. **a**, **b** are CPD staining pattern (red signals) and 45S rDNA-FISH signals (red) of chromosomes in *L. radiata*, respectively. **c**, **d** are CPD pattern and 45S rDNA-FISH signals (red) of *L. aurea*. **e**, **f** and **g**, **h** are the hybrid 1 and 2, respectively. Scale bars: 10 μm. Five cells in five different individuals of each sample were detected

**Fig. 3:**
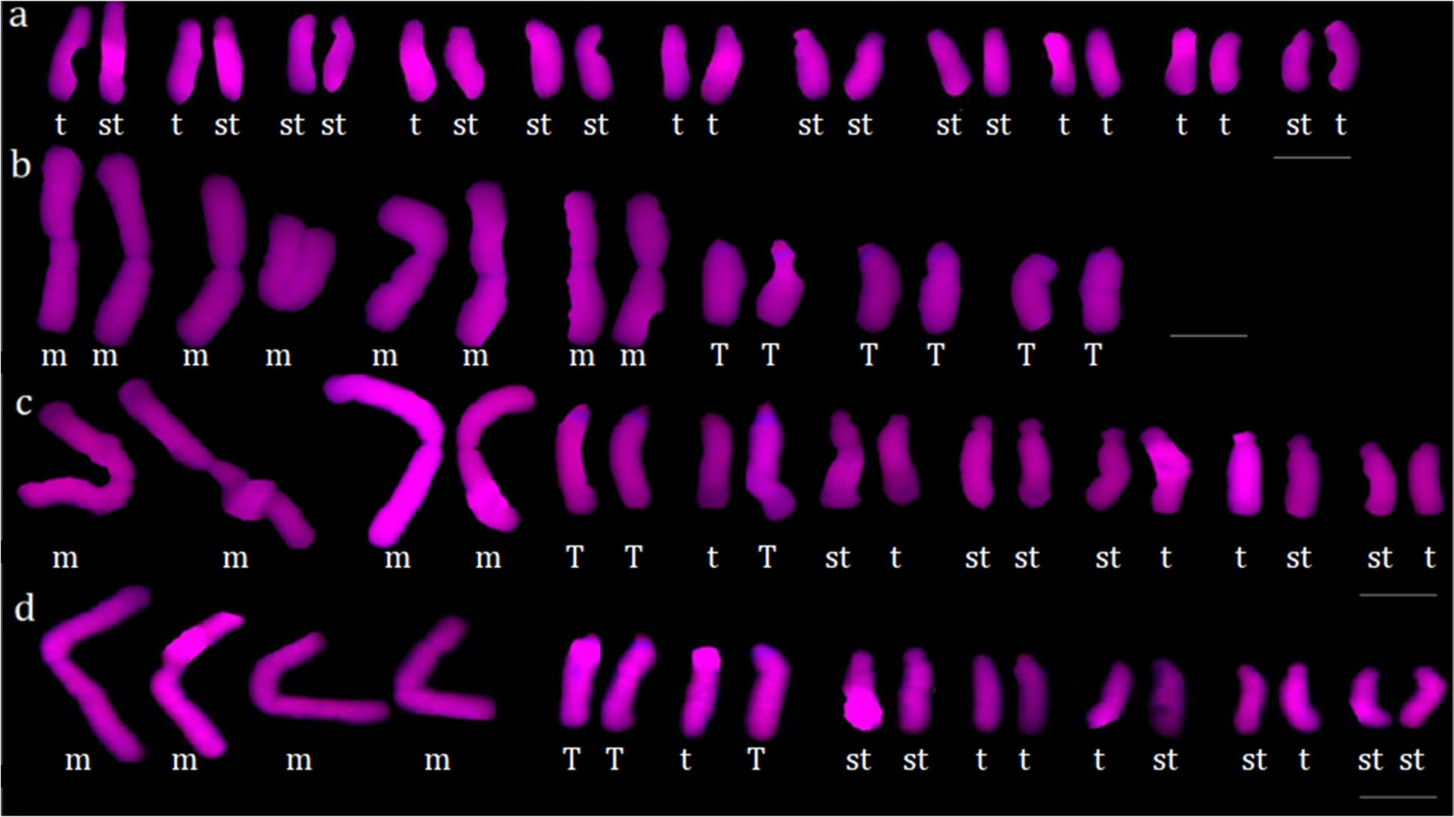
The chromosome morphology and karyotypes of *L. radiata* (**a**), *L. aurea* (**b**), the hybrid 1 (**c**) and the hybrid 2 (**d**). T Terminal centromeric point. t terminal centromeric region. st subterminal centromeric region. m median centromeric region. Scale bars: 10 μm. Five cells in five different individuals of each sample were detected

CPD staining patterns and 45S rDNA-FISH signals in four *Lycoris* species were shown in Fig. 2, and their karyotype features including chromosome morphology, karyotype, CPD staining bands, DAPI bands, and 45S rDNA-FISH bands were shown in Fig. 3 and Table 2.

**Table 2:** Karyotype features of four taxa in *Lycoris*

Specie	Chromosome		CPD bands		DAPI bands		FISH bands		AT	A_1_	A_2_	TCL (μm)
	2n	Karyotype	Number	Position	Number	Position	Number	Position				
*L. radiata*	22	10t + 12st	4	S-TERs of four t/st	0	-	4	S-TERs of four t/st	4A	0.811	0.063	115.61
*L. aurea*	14	8 m + 6 T	6	CENs of six T	6	PCENs of six T	6 + 2 + 2	CENs of six T; PCENs of two T; CENs of two m	2B	0.438	0.041	145.95
Natural hybrid 1	18	4 m + 6st + 5t + 3 T	5	CENs of fiveT/t/st	3	PCENs of three T	5 + 1 + 1	CENs of five T/t/st; PCEN of one T; CEN of one m	3B	0.719	0.052	128.88
Natural hybrid 2	18	4 m + 6st + 5t + 3 T	5	CENs of five T/t/st	3	PCENs of three T	5 + 1 + 1	CENs of five T/t/st; PCEN of one T; CEN of one m	3C	0.723	0.030	129.69

*T* Terminal centromeric point. *t* terminal centromeric region. *st* subterminal centromeric region. *m* median centromeric region. *S* short arm. *CEN* centromeric. *PCEN* pericentromeric. *TER* terminal. *AT* Stebbins’ karyotype asymmetry type. *A*_*1*_ intrachromosomal asymmetry index. *A*_*2*_ interchomosomal asymmetry index. *TCL* average haploid total chromosome length. Five cells in five different individuals of each sample were detected

The karyotype parameters of *L. radiata* were shown in Supplementary Table S1. Its chromosomes were 2n = 22, including 12 st-type and 10 t-type chromosomes (Fig. 3a), and the ratio of the lengest/shortest chromosome(L/S) was 1.73. The average haploid total chromosome length (TCL) was 115.61 μm. The values of intrachromosomal asymmetry index (A_1_) and interchomosomal asymmetry index (A_2_) were 0.811 and 0.063, respectively. Its karyotype formula was 2n = 2x = 22 = 10t + 12st, with 4A Stebbins’ karyotype asymmetry type. As shown in Fig. 2a, four red CPD bands in the terminal regions of short arms of chromosome 3, 4, 9 and 10 were detected respectively, but positive DAPI signals were absent in the chromosomes of *L. radiata*. Similarly, four red 45S rDNA-FISH signals (Fig. 2b) in the same regions of the four chromosomes were detected respectively, indicating that the CPD staining regions were also 45S rDNA-FISH signal sites in *L. radiata*.

The karyotype parameters of *L. aurea* were shown in Supplementary Table S2. Its chromosomes were 2n = 14, including 8 m-type and 6 T-type chromosomes (Fig. 3b), with the L/S ratio 2.45. The TCL was 145.95 μm. The values of A_1_ and A_2_ were 0.438 and 0.041, respectively. Its karyotype formula was 2n = 2x = 14 = 8 m + 6 T, 2B type. As shown in Fig. 2c, each of six T-type chromosomes had a red CPD band in the terminal centromeric point of chromosome 9, 10, 11, 12, 13 and 14, and also possessed a positive DAPI band in every pericentromeric regions of the six chromosomes respectively. At the same time, six red 45S rDNA-FISH signals stained in the same points of the six T-type chromosomes were detected respectively (Fig. 2d). Besides, four red 45S rDNA-FISH signals were also found in the median centromeric regions of a pair of m-type homologous chromosome 7, 8, and in the pericentromeric regions of a pair of T-type homologous chromosome 13, 14, respectively.

The karyotype parameters of natural hybrid 1(*L*. *hunanensis*) were shown in Supplementary Table S3. Its chromosomes were 2n = 18, including 4 m-, 3 T-, 6 st-, and 5 t-type chromosomes (Fig. 3c), with the L/S ratio 3.50. The TCL was 128.88 μm. The values of A_1_ and A_2_ were 0.719 and 0.052, respectively. Its karyotype formula was 2n = 2x = 18 = 4 m + 6st + 5t + 3 T, 3B type. As shown in Fig. 2e, each of chromosome 5, 6, 7, 8 and 9 had a red CPD band in the centromeric points/regions of them respectively, in which each of T-type chromosome 5, 6 and 8 possessed a positive DAPI band in the pericentromeric region respectively. As was known from Fig. 2f, each of the five chromosomes from 5 to 9 had a red 45S rDNA-FISH signal in the centromeric point/region respectively, and also the FISH signals were also detected in the pericentromeric region of the chromosome 5 (T-type), as well as in the median centromeric region of the chromosome 1 (m-type), respectively.

The karyotype parameters of natural hybrid 2 were shown in Supplementary Table S4. Its chromosomes were also 2n = 18, including 4 m-, 3 T-, 6 st-, and 5 t-type chromosomes (Fig. 3d), with the L/S ratio 4.31. The TCL was 129.69 μm. The values of A_1_ and A_2_ were 0.723 and 0.030, respectively. Likewise, its karyotype was also formulated as 2n = 18 = 4 m + 6st + 5t + 3 T, 3C type. As shown in Fig. 2g and h, the karyotype features of the hybrid 2, including CPD bands, DAPI bands and 45S rDNA-FISH signals etc., were as same as those of the hybrid 1 respectively. For example, each of the five chromosomes from 5 to 9 had a red CPD band in the centromeric points/regions respectively, in which the three T-type chromosomes also possessed a positive DAPI band in the pericentromeric regions respectively, etc. The other karyotype features of the hybrid 2 were displayed in Table 2 and would not be described here.

### Chloroplast genomes sequencing and analysis

#### Basic feature of the four chloroplast genomes in *Lycoris*

**Fig.4:**
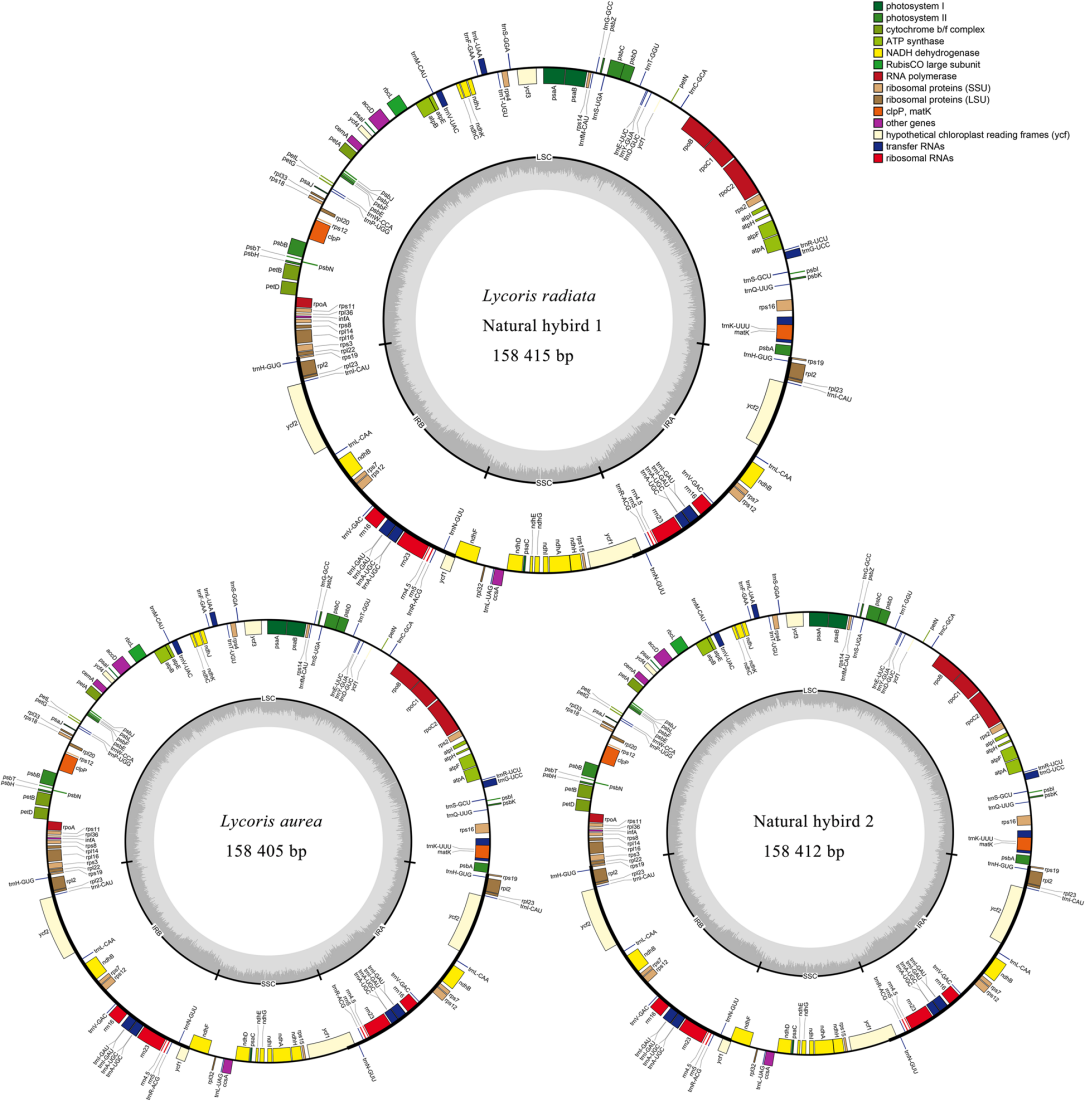
Chloroplast genome map of the four samples in *Lycoris*

The circular chloroplast genomes of the four *Lycoris* species were 158,405–158415 bp in size (Fig. 4), consisting of a pair of inverted repeat (IR, 26,733 bp), separated by large single copy (LSC, 86,596–86,598 bp) and small single copy (SSC, 18,342–18,351 bp) regions. Of these, the size of *L. radiata* and natural hybrid 1 (*L. hunanensis*), including LSC, SSC, IR and protein coding gene (PCG) regions, was the same, but they were slightly different from *L. aurea* and natural hybrid 2 in these regions (Table 3). Their cp genomes all contained 137 genes, including 87 PCGs, 42 tRNA genes and 8 rRNA genes. These genes of the four samples were completely consistent in functional classification, including 44 photosynthesis related genes (e.g. *rbc*L, *ndh*A, *atp*A, etc.), 80 transcription and translation related genes (e.g. *rpl*2, *rps*2, *rpo*A, *rrn*5, *trn*A-UGC, etc.), 6 others genes (e.g. *mat*K, *acc*D, *ccs*A, etc.), and 7 unknown function gene such as *ycf*1, *ycf*2, etc. Of these genes, 26 genes (e.g. *pet*B, *atp*F, *ndh*A, etc.) and 2 genes (*ycf*3, *clp*P) contained 1 and 2 introns respectively in the four *Lycoris* cp genomes (Table 4), suggesting the high conservation of chloroplast genes in the genus.

**Table 3:** Comparison of the genome characters of the four *Lycoris* chloroplasts

Species	Size	Genome size(bp)				Number of genes				GC (%)
		LSC	SSC	IR	PCG	PCG	tRNA	rRNA	Total	
*L. aurea*	158405	86597	18342	26733	79344	87	42	8	137	37.81
Natural hybrid 1	158415	86598	18351	26733	79422	87	42	8	137	37.80
Natural hybrid 2	158412	86596	18350	26733	79428	87	42	8	137	37.80
*L. radiata*	158415	86598	18351	26733	79422	87	42	8	137	37.80

Five plants of each sample were selected to analyse chloroplast genomes

*LSC* large single copy, *SSC* small single copy. *IR* inverted repeat. *PCG* protein coding gene

**Table 4:** Functional classification of chloroplast genes of four samples in *Lycoris*

Classification	Gene family	Gene name
Photosynthesis related genes (44)	Photosystem I	*psa*A, *psa*B, *psa*C, *psa*I, *psa*J
	Photosystem II	*psb*A, *psb*B, *psb*C, *psb*D, *psb*E, *psb*F, *psb*H,*psb*I, *psb*J, *psb*K, *psb*L, *psb*N,*psb*T, *psb*Z
	Cytochrome b/f complex	*pet*A, *pet*B^※^, *pet*D^※^, *pet*G, *pet*L, *pet*N
	ATP synthase	*atp*A, *atp*B, *atp*E,*atp*F^※^, *atp*H, *atp*I
	NADH plastoquinone oxidoreductase	*ndh*A^※^, *ndh*B^a,^^※^, *ndh*C, *ndh*D, *ndh*E, *ndh*F^※^, *ndh*G, *ndh*H, *ndh*I, *ndh*J, *ndh*K
	ribulose-1,5-bisphosphate carboxylase	*rbc*L
Transcription and translation related genes (80)	rRNA gene	*rrn*4.5^a^, *rrn*5^a^, *rrn*16^a^, *rrn*23^a^
	tRNA gene	*trn*A-UGC^c, ※^, *trn*C-GCA, *trn*D-GUC, *trn*E-UUC, *trn*F-GAA, *trn*G-UCC^※^, *trn*G-GCC,*trn*H-GUG^a^, *trn*I-GAU^c^,*trn*I-CAU^a^, *trn*K-UUU^※^, *trn*L-CAA^a^, *trn*L-UAA^※^, *trn*L-UAG, *trn*M-CAU, *trn*fM-CAU,*trn*N-GUU^a^, *trn*P-UGG, *trn*Q-UUG, *trn*R-ACG^a^, *trn*R-UCU, *trn*S-GGA, *trn*S-GCU, *trn*S-UGA, *trn*T-GGU, *trn*T-UGU, *trn*V-UAC^※^,*trn*V-GAC^a^, *trn*W-CCA, *trn*Y-GUA
	Small subunit of ribosome	*rps*2,*rps*3,*rps*4, *rps*7^a^, *rps*8, *rps*11, *rps*12^a,^^※^, *rps*14,*rps*15, *rps*16^※^, *rps*18, *rps*19^a^
	Large subunit of ribosome DNA dependent RNA polymerase	*rpl*2^a,^^※^, *rpl*14, *rpl*16^※^, *rpl*20, *rpl*22,*rpl*23^a^, *rpl*32, *rpl*33, *rpl*36 *rpo*A, *rpo*B, *rpo*C1^※^, *rpo*C2
Others genes (6)	Maturase	*mat*K
	Acetyl-CoAcarboxylase	*acc*D
	Cytochrome C heme attachment protein	*ccs*A
	Chloroplast envelop membrane protein	*cem*A
	Clp protease	*clp*P^※※^
	Translational initiation factor	*inf*A
Unknown function genes (7)	Hypothetical chloroplast conserved open reading frames	*ycf*1^b^, *ycf*2^a^, *ycf*3^※※^, *ycf*4

The number of genes in parentheses

^a, b, c^represent the gene has two, three and four, respectively. ^※^, ^※※^ represents the gene contains one, two introns, respectively. Five plants of each sample were selected to analyse chloroplast genomes

### Comparisons of IR-LSC/SSC boundaries in the four *Lycoris* taxa

**Fig. 5:**
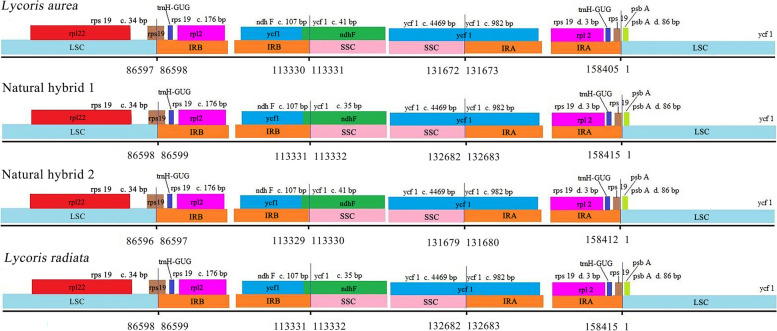
Comparison of the borders of large single copy (LSC), small single copy (SSC), and inverted repeat (IR) regions among 4 *Lycoris* germplasm chloroplast genomes. **c** means distance cross over IR border. **d** means distance from IR border in this picture

The circular structures of the cpDNA genomes of the four *Lycoris* taxa made four boundaries among LSC, SSC and IR, which were LSC-IRB, IRB-SSC, SSC-IRA, and IRA-LSC (Fig. 5). As shown in Fig. 5, among the four cp genomes, the genes and shrinkages or expansions of the IR boundaries were the same at the LSC-IRB, SSC-IRA and IRA-LSC borders. For example, the LSC-IRB borders of the four *Lycoris* samples were within the *rps*19, where the gene was 34 bp and 176 bp in the IRB and LSC regions respectively, and the IRA-LSC borders of them were all between the *rps*19 and *psb*A, their distances from the borders being 3 bp and 86 bp respectively. The IRB-SSC boundaries of the four samples were on the *ndh*F and *ycf*1, 107 bp *ndh*F spanned the IRB-SSC regions duplicated at the IRB regions in the four species, but there were minor differences at the *ycf*1 gene of the IRB-SSC regions of the four genomes, namely, 41 bp *ycf*1 of the IRB regions duplicated at the SSC regions in *L. aurea* and natural hybrid 2, 35 bp of the gene duplicated in *L. radiata* and natural hybrid 1 respectively. Therefore, among the four *Lycoris* taxa, the genes and features of the IR boundaries were the completely same between *L. aurea* and natural hybrid 2, *L. radiata* and natural hybrid 1 respectively, suggesting that the putative natural hybrid 1 and hybrid 2 were more recently related to *L. radiata* and *L. aurea*, respectively.

### SSR analysis

The chloroplast simple sequence repeats (cp SSRs) of the four *Lycoris* taxa were analyzed in this study. The numbers and types of the cp SSRs in these samples were shown in Supplementary Table S5. There were 58, 58, 63, and 63 SSRs in the *L. radiata*, natural hybrid 1, *L. aurea*, and natural hybrid 2 cp genomes, respectively. Six types of nucleotide repeats, including mononucleotide, di-nucleotide, tri-nucleotide, tetra-nucleotide, penta-nucleotide and compound sequence, were detected in the four *Lycoris* taxa. Among the different unit sizes, the mononucleotide was the most frequent, accounting more than 55% of all types in the samples, in which base T and A were the primary elements, only one C motif in *L. aurea* and natural hybrid 2, and two C in *L. radiata* and natural hybrid 1, but no G in the four samples. The numbers and types of the cp SSRs in *L. radiata* and the hybrid 1 were almost the same except for one different mononucleotide repetition, i.e. the former "(A) 10" and the latter "(A) 11" in the start site 30,544 bp, while only two repetitions differed in these SSR characteristics of *L. aurea* and the hybrid 2, including the mononucleotide repetition "(T) 14" and "(T) 13" in the start site 23,149 bp, the penta-nucleotide "(GGAAA)3" and "(CGAAA)3" in the start site 111,168 bp, respectively. However, these SSR characteristics of the hybrid 2 were significantly different compared with *L. radiata*. and the hybrid 1. For example, nine mononucleotide repetitions of cp SSRs in *L. aurea* and the hybrid 2, including “(T)11” (3701 bp) and “(A)10” (8381 bp) etc., were missing in *L. radiata* and the hybrid 1, respectively. At the same time, six mononucleotide repetitions of *L. radiata* and the hybrid 1, including “ (A) 11” (14,137 bp) and “(A)10”(33,464 or 33,465 bp) etc., were missing in *L. aurea* and the hybrid 2, respectively. Therefore, the results suggested that the putative natural hybrid 1 and hybrid 2 were more recently related to *L. radiata* and *L. aurea*, respectively.

### SNV analysis

To reveal the differences of cp genomes in the four *Lycoris* species, interspecific comparisons were performed using *Lycoris radiata* (MN158120) plastome previously reported as the reference sequence (Table 5). As shown in Table 5, there were 150, 115, 151, and 138 SNPs in *L. aurea,* natural hybrid 1, hybrid 2, and *L. radiata* respectively, a total of 190 SNPs in the four samples. Of these SNPs, there were 36 and 120 same SNP sites between *L. aurea* and the hybrid 1, *L. aurea* and the hybrid 2 respectively, 103 and 30 same SNP sites between *L. radiata* and the hybrid 1, *L. radiata* and the hybrid 2 respectively. There were significant differences in the variation sites of the four cp genomes, including 50–82 SNPs located in 26–29 genes, 6–9 short insertions and 11–22 short missing fragments. Compared to the control (MN158120), the bases CA (113,368–113,369 bp) and GT (58,741–58,742 bp) were inserted in the *ycf*1 gene of *L. aurea* and *rbc*L gene of *L. radiata* respectively, but not changed in these genes of the hybrid 1; the base G was mutated to A (78,820 bp) in the gene *pet*B of *L. radiata* and the hybrid 1. Hence, these results showed that sequence variations between *L. radiata* and the hybrid 1 (*L. hunanensis*), *L. aurea* and the hybrid 2 were minor respectively, indicating the affinities and distances among the four *Lycoris* species.

**Table 5:** Chloroplast genome variation sites of four samples in *Lycoris*

Variation types	*L. aurea*	Natural hybrid 1	Natural hybrid 2	*L. radiata*	Total sites	Number of same SNP sites			
						*L. aurea* and hybrid 1	*L. aurea* and hybrid 2	*L. radiata* and hybrid 1	*L. radiata* and hybrid 2
SNP	150	115	151	138	190	36	120	103	30
SNP located genes	75(27)	50(27)	82(26)	72(29)	112(32)				
Insert	6(1)	8(0)	9(2)	9(1)	10(2)				
Missing	20(6)	11(2)	22(6)	11(3)	20(6)				

Numbers in parentheses mean variation number of genes

### Phylogenetic analysis

**Fig. 6:**
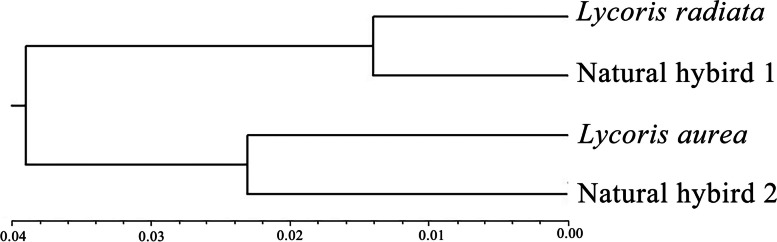
UPGMA dendrogram based on average Euclidean distances among the four *Lycoris* species

The UPGMA dendrogram of the four *Lycoris* species was generated based on average Euclidean distances in this study (Fig. 6). As shown in Fig. 6, dissimilarity values between taxa ranged from 0.014 to 0.039. Of these *Lycoris* species, *L. radiata* and putative natural hybrid 1 (*L. hunanensis*) were clustered into a clade while *L. aurea* and natural hybrid 2 were clustered into the other clade, indicating that their relationships between *L. radiata* and natural hybrid 1, *L. aurea* and natural hybrid 2 were much closer, respectively.

**Fig. 7:**
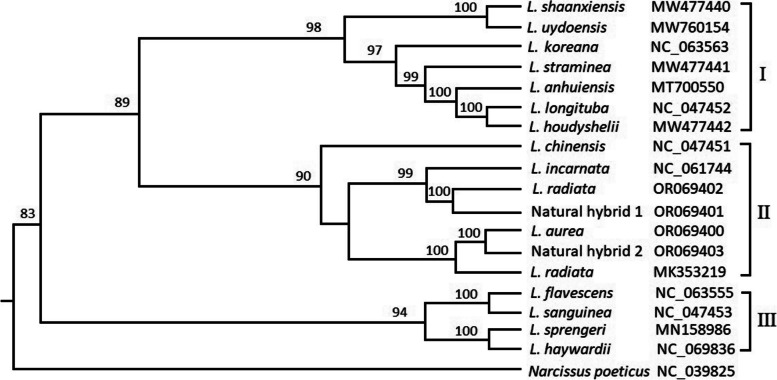
Phylongenetic tree by maximum likelihood analysis based on complete cp genomes

In order to explore the interspecific relationships of the four samples and phylogenetic relationships of *Lycoris* species, the phylongenetic tree was constructed by maximum likelihood analysis based on complete cp genomes of 18 *Lycoris* species (Fig. 7). *Narcissus poeticus* (NC_039825) was selected as outgroup in this study. As shown in Fig. 7, 18 *Lycoris* species were clustered into three clades, *Narcissus poeticus* (outgroup) formed an alone branch. Among these samples, *L. aurea*, *L. radiata* (OR069402)*, L. radiata* (MK353219), two natural hybrids, *L. incarnate* and *L. chinensis* were clustered into the second clade, indicating that the seven *Lycoris* species had a closer relationships in *Lycoris*. Especially between *L. radiata* and natural hybrid 1 (*L. hunanensis*), *L. aurea* and natural hybrid 2 could be divided into a minor branch in the second clade respectively, suggesting that interspecific relationships between *L. radiata* and natural hybrid 1 (*L. hunanensis*), *L. aurea* and natural hybrid 2 were much closer respectively. Obviously, the clusters of the four *Lycoris* species derived from the phenetic and chloroplast genomic data respectively were completely consistent, sufficiently indicating that the result of their interspecific relationships was correct and reliable.

## Discussion

The karyotype evolution and phylogenetic relationship of species were complex and ambiguou, but some mechanisms, including Robertsonian fusion and fission, as well as hybridization etc., were well accepted [7, 21–23]. Hybridization and polyploidization were considered as important models of speciation in this genus [6, 17, 24, 25]. In the phylogenetic tree, *L. flavescens* (NC_063555), *L. sanguine* (NC_047453), *L. sprengeri* (MN158986) and *L. haywardii* (NC_069836) were clustered into the third clade, furthermore, the first two and the latter two could be divided into a minor branch respectively, indicating that they had a closer relationships in *Lycoris.* Thus, hybrid origins about *L. haywardii* and *L. flavescens* etc. [16, 17] were supported by chloroplast genome evidence. Of 18 *Lycoris* species, *L. aurea,* natural hybrid 2 and *L. radiata* (MK353219) could be divided into a minor branch in the second clade, suggesting that interspecific relationships of the three taxa were much closer in *Lycoris*. *L. straminea* was considered a descendant of *Lycoris chinensis* and *Lycoris radiata* var. *pumila* [1, 26, 27]. *L. straminea* (2n = 19) and *L. hunanensis* (2n = 18) were of similar species based on morphological features such as leaf shape and flower colour etc. [20], however, they were clustered into different clades in *Lycoris*, indicating that there was obvious differences between morphology and cp genomes classification.

Theoretically, *L. radiata* (2n = 22 = 10t + 12st) and *L. aurea* (2n = 14 = 8 m + 6 T) could produce gametes with the karyotype compositions (*n* = 11 = 5t + 6st) and (*n* = 7 = 4 m + 3 T) by the meiosis respectively, and the F_1_ progeny with the karyotype (2n = 18 = 4 m + 6st + 5t + 3 T) was produced by the fertilization uniting the male and female gametes. In our study, the two hybrid progenies with the karyotype composition (2n = 18 = 4 m + 6st + 5t + 3 T) were found in natural habitats (Table 2) and the representative heterozygosity features of karyotype were detected and affirmed, including four karyotypes (m, st, t, and T), 18 chromosomes etc., indicating that the two variant taxa of *Lycoris* originated from their hybridization of *L. radiata* and *L. aurea* in terms of karyotype. Judging from the absence of mature fruits and seeds, the two hybrid generations were sterile, which might be related to their compound karyotype compositions with m-, st-, t-, and T-type chromosomes, causing the failure of chromosomes pairing properly at meiosis [28].

This study showed that the corresponding chromosome features in the two hybrids, including special CPD bands and 45S rDNA-FISH signals etc., were all found in the chromosomes of their diploid parents (*L. radiata* and *L. aurea*). For example, 2 t/st-type chromosomes labeled by red CPD staining bands (one per chromosome) and especially only one m-type chromosome (number 1 in Fig. 2f and number 4 in Fig. 2h) labeled by a 45S rDNA-FISH signal (red) at the same sites in the two hybrids respectively, separated from a pair of m-type chromosomes (number 7 or 8 in Fig. 2d) of diploid parent (*L. aurea*), were detected in our study. And also three T-type chromosomes from their parent (*L. aurea*), with the positive DAPI signals in the two hybrids, were all detected at the same points (Table 2), suggesting that the putative natural hybrid 1 and the hybrid 2 possessed the heterozygosity features of the diploid parents (*L. radiata* and *L. aurea*), which were completely supported by the cp genome sequence analysis in this study (Figs. 4, 5, 6; Tables 3, 4, 5). In general, these results in this study strongly supported that natural hybridization was an important model of species origin and karyotype evolution in *Lycoris*.

The chloroplast genome was relatively stable and genetically conserved because it possessed an independent genome from the mother, and played an indispensable role in elucidating interspecific relationship etc. [29–31]. In this study, the results showed that a total of 137 genes were all annotated, including 87 PCGs, 42 tRNAs, and eight rRNAs, with 158,405–158415 bp sizes of the complete cp genomes in *L. aurea*, *L. radiata*, the putative natural hybrid 1 and the hybrid 2 (Table 3). These were consistent with the previous studies about cp genomes (137 genes) of *L. chinensis*, *L. anhuiensis*, and *L. aurea* reported by zhang et al. [8], suggesting that the cp genomes of *Lycoris* were highly conserved in structure. However, there was a significant difference from the 127 genes of the *L. aurea* chloroplast genome reported by Peng et al. [32], explaining that there was obvious variations of cp genomes among different populations in *L. aurea*. Of the four samples, compositions and structures of the chloroplast genomes in *L. radiata* and the putative natural hybrid 1, including their basic features, boundary genes, SSRs, and SNPs etc. (Table 5), were almost the same, suggesting that genetic relationship between the two species was closely related. Combining the karyotype analysis results in this study, we affirmed that the putative natural hybrid 1 (*L. hunanensis*) originated from the natural hybridization of *L. radiata* (♀) × *L. aurea* (*♂*). Similarly, the composition and structure features of the chloroplast genomes in *L. aurea* and the putative natural hybrid 2 were almost the same (Table 5, Fig. 5), suggesting their closer genetic relationship. Similarly, we also affirmed that the putative natural hybrid 2 originated from the hybridization of *L. radiata* (*♂*) × *L. aurea* (♀).

## Conclusion

We found strong evidences for reciprocal natural hybridization between *L. radiata* (2n = 22) and *L. aurea* (2n = 14) by fluorescence in situ hybridization (FISH) and high-throughput sequencing etc., which gave rise to two new taxa (2n = 18) of *Lycoris* including the putative natural hybrid 1 (*L. hunanensis*) and hybrid 2, possessing their parental heterozygosity features in morphology, karyotype and chloroplast genome. This study revealed the origin of two new species of *Lycoris* and strongly supported the role of natural hybridization that facilitated lineage diversification in this genus.

## Materials and methods

### Plant material

*Lycoris radiata* (L’Her.) Herb. (Fig. 1a, voucher number HHUL004), *Lycoris aurea* (L’Her.) Herb. (Fig. 1b, voucher number HHUL001), putative natural hybrid 1 (*L. hunanensis*) (Fig. 1c-e, voucher number HHUL007) and hybrid 2 (Fig. 1f-h, voucher number HHUL010) were collected from the same distribution area in Yuanling County, Hunan Provence, China. These plant materials were cultivated in the botanical garden of Huaihua University for follow-up studies.

### Measurement of major morphological traits

The measurements of the morphological traits were conducted in different growth and development periods because the special feature of *Lycoris* is the absence of leaves while flowering. The morphological parameters of these samples, including flower colors in different anthesis and leaves (e.g. length, width) etc., were measured in August and December of the same year, respectively. Flower colors were quantified according to the standard of international color card. Five healthy and strong plants (three leaves each plant) from each sample were randomly selected to determine morphological traits, averaged and standard deviations were calculated in this study.

### Karyotype analysis

#### CPD staining and fluorescence in situ hybridization (FISH) of the chromosomes

The chromosome preparations of the four *Lycoris* species and CPD staining were performed as previously described [12] with minor modification in this study. In brief, the root tips were fixed in methanol: acetic acid (3:1) for 3 h after treatment with the *α*-bremnaphthalene at 28 ℃ for 4 h, and macerated with an enzyme mixture at 28 ℃ for 3 h. The well-spread chromosome preparations were used to perform CPD staining and FISH [33]. The chromosome preparations were stained with 4’, 6-diamidno-2-phenylindole (DAPI) etc.. The chromosomes and hybridization signals were observed by Olympus BX60 fluorescence microscope etc.

### Measurements of karyotype parameters

The karyotype parameters of the four samples, including the relative length of short arm (SL) and long arm (LL), total relative length (TL), arm ratio (LL/SL), Stebbins’karyotype asymmetry type (AT), total chromatin length (TCL) and karyotype assymetry indices (A_1_, A_2_) etc. were obtained according to the methods [22, 34–38]. For each sample analyzed in this study, measurements were taken from at least five metaphase cells in five different individuals. The karyotypes included T-type (terminal centromeric point, arm ratio ∞), t-type (terminal centromeric region, arm ratio > 7.00), st-type (subterminal centromeric region, arm ratio 3.01–7.00), and m-type (median centromeric region, arm ratio 1.01–1.70) [12, 13].

### Chloroplast genome sequencing and ananlysis

#### Sample collection, DNA extraction, and sequencing

Five healthy and strong plants from each sample were randomly selected to analyse chloroplast genomes in this study.The fresh leaves of the four samples in *Lycoris* were collected, flash frozen in liquid nitrogen, and stored at − 80 ℃ for DNA extraction. Genomic DNA was isolated using the Plant Genomic DNA Kit (Shanghai, China) according to the instructions, and its quality was examined using NanoDrop 2000 (Thermo Fisher Scientific, USA) etc. High-quality DNA was used for libraries’ construction and sequencing, which was sequenced at Illumina Hiseq 2500 (Illumina, USA) using 2 × 150 two-end sequencing strategy with an insert size of 300 bp for high-throughput sequencing etc.

### cpDNA genome sequences assembly, annotation and structure analysis

The chloroplast DNA (cpDNA) genome sequences of the four samples were assembled and annotated using the softwares such as metaSPAdes 3.13.0, ogdraw 1.1.1 etc. The cp genome sequences of *L. aurea*, *L. radiata*, putative natural hybrid 1 (*L. hunanensis*), and natural hybrid 2 were deposited into GenBank for the first time with accession numbers OR069400, OR069402, OR069401, and OR069403, respectively. The complete cpDNA sequence of *Lycoris radiata* (MN158120) was selected as a reference [39] in order to obtain the complete annotation results. The physical maps of the cpDNA genomes of the *Lycoris* samples were drawn using software OGDRAW [8].

### Interspecific genome comparison

The cpDNA genome sequences of the four *Lycoris* samples were analyzed using Mummer 3.0 in order to identify large single-copy region (LSC), small single-copy region (SSC) etc. The chloroplast simple sequence repeats (cpSSR) of the genomes were identified using MISA. The inverted repeats (IR) on the boundary of junction sites and single nucleotide variants (SNV) etc. were identified or analyzed using bwa (0.7.17) and gatk (4.0.8.1) etc. [8, 40, 41].

### Phylogenetic analysis

In the four *Lycoris* species, a phenetic analysis was performed by using the eight variables per species: leaf (length and width), flower color (bud, early anthesis and mid-anthesis) and chromosome length (A_1_, A_2_ and TCL). The data matrix of the variables was standardized and average Euclidean distance was calculated using INFOSTAT version 1.1. The UPGMA dendrogram of these taxa was generated based on the morphometric and karyological data [37, 38].

The four complete cpDNA sequences of *Lycoris* were obtained in this study, and fourteen cp sequences of *Lycoris*, including *L. shaanxiensis* (MW477440), *L. uydoensis* (MW760154), *L. koreana* (NC_063563), *L. straminea* (MW477441), *L. anhuiensis* (MT700550), *L. longituba* (NC_047452), *L. houdyshelii* (MW477442), *L. chinensis* (NC_047451), *L. incarnate* (NC_061744), *L. radiata* (MK353219), *L. flavescens* (NC_063555), *L. sanguine* (NC_047453), *L. sprengeri* (MN158986) and *L. haywardii* (NC_069836), were downloaded from NCBI. *Narcissus poeticus* (NC_039825) was selected as the outgroup. Both the complete cp genome sequences and genes were used for tree construction. Maximum likelihood phylogenies based on the best-fit model was conducted using orthofinder (2.2.7) and ggtree (1.14.6). The best-fit model was estimated using 1000 bootstrap replicates [8, 42].

### Supplementary Information

**Additional file 1: Supplementary Table S1.** Measurements of somatic chromosomes of *L. radiata*. **Supplementary Table S2.** Measurements of somatic chromosomes of *L. aurea*. **Supplementary Table S3.** Measurements of somatic chromosomes of natural hybrid 1. **Supplementary Table S4.** Measurements of somatic chromosomes of natural hybrid 2. **Supplementary Table S5.** The chloroplast genome SSR loci distributions of four samples in *Lycoris*.

## Data Availability

The data that support the results are included in this article and its supplementary materials. The raw sequencing data of the chloroplast genome sequences have been deposited in NCBI (https://www.ncbi.nlm.nih.gov/) with accession number: OR069400, OR069401, OR069402 and OR069403.
