# Supplementary material for: Reciprocal natural hybridization between Lycoris aurea and Lycoris radiata (Amaryllidaceae) identified by morphological, karyotypic and chloroplast genomic data
Source: BMC Plant Biol. 2024 Jan 2;24:14. doi: 10.1186/s12870-023-04681-2 (PMC10759762; doi:10.1186/s12870-023-04681-2)
Supplement: Supplementary file 1 — Additional file 1: Supplementary Table S1. Measurements of somatic chromosomes of L. radiata. Supplementary Table S2. Measurements of somatic chromosomes of L. aurea. Supplementary Table S3. Measurements of somatic chromosomes of natural hybrid 1. Supplementary Table S4. Measurements of somatic chromosomes of natural hybrid 2. Supplementary Table S5. The chloroplast genome SSR loci distributions of four samples in Lycoris. [file 12870_2023_4681_MOESM1_ESM.doc]

**Supplementary data**

**Supplementary Table S1** Measurements of somatic chromosomes of *L. radiata*

**Supplementary Table S2** Measurements of somatic chromosomes of *L. aurea*

**Supplementary Table S3** Measurements of somatic chromosomes of natural hybrid 1

**Supplementary Table S4** Measurements of somatic chromosomes of natural hybrid 2

**Supplementary Table S5** The chloroplast genome SSR loci distributions of four samples in *Lycoris*

**Supplementary Table S1** Measurements of somatic chromosomes of *L. radiata*

| No. | Relative length (%) | | | Arm ratio (LL/SL) ±SD | Type |
| --- | --- | --- | --- | --- | --- |
|  | LL±SD | SL±SD | TL±SD |  |  |
| 1 | 5.19±0.35 | 0.63±0.05 | 5.82±0.39 | 8.24±0.45 | t |
| 2 | 5.25±0.21 | 0.82±0.06 | 6.07±0.26 | 6.40±0.31 | st |
| 3 | 4.45±0.28 | 0.60±0.04 | 5.05±0.32 | 7.42±0.35 | t |
| 4 | 4.15±0.25 | 0.88±0.05 | 5.03±0.31 | 4.71±0.26 | st |
| 5 | 3.98±0.22 | 0.99±0.06 | 4.97±0.27 | 4.02±0.30 | st |
| 6 | 3.63±0.19 | 1.21±0.08 | 4.84±0.28 | 3.00±0.20 | st |
| 7 | 4.59±0.30 | 0.36±0.01 | 4.95±0.32 | 12.75±0.58 | t |
| 8 | 3.71±0.24 | 1.04±0.09 | 4.75±0.32 | 3.57±0.21 | st |
| 9 | 3.63±0.23 | 1.18±0.08 | 4.81±0.31 | 3.08±0.23 | st |
| 10 | 3.68±0.20 | 0.96±0.05 | 4.64±0.26 | 3.83±0.21 | st |
| 11 | 3.98±0.33 | 0.55±0.04 | 4.53±0.39 | 7.24±0.41 | t |
| 12 | 4.12±0.26 | 0.58±0.03 | 4.70±0.28 | 7.10±0.29 | t |
| 13 | 3.60±0.28 | 0.85±0.05 | 4.45±0.32 | 4.24±0.22 | st |
| 14 | 3.43±0.25 | 0.82±0.08 | 4.25±0.29 | 4.18±0.23 | st |
| 15 | 3.19±0.17 | 0.96±0.06 | 4.15±0.22 | 3.32±0.19 | st |
| 16 | 3.09±0.24 | 1.02±0.07 | 4.11±0.30 | 3.03±0.24 | st |
| 17 | 3.63±0.15 | 0.25±0.01 | 3.88±0.17 | 14.52±0.71 | t |
| 18 | 3.52±0.11 | 0.47±0.03 | 3.99±0.12 | 7.49±0.56 | t |
| 19 | 3.68±0.31 | 0.33±0.02 | 4.01±0.30 | 11.15±0.67 | t |
| 20 | 3.35±0.29 | 0.27±0.02 | 3.62±0.32 | 12.41±0.81 | t |
| 21 | 2.88±0.17 | 0.63±0.05 | 3.51±0.24 | 4.57±0.32 | st |
| 22 | 3.57±0.26 | 0.3±0.04 | 3.87±0.29 | 11.90±0.68 | t |

**LL** relative length of long arm. **SL** relative length of short arm. **TL** total relative length. **SD** standard deviation. Five cells in five different individuals of each sample were detected. The same below.

**Supplementary Table S2** Measurements of somatic chromosomes of *L. aurea*

| No. | Relative length (%) | | | Arm ratio (LL/SL) | Type |
| --- | --- | --- | --- | --- | --- |
|  | LL±SD | SL±SD | TL±SD |  |  |
| 1 | 4.76±0.22 | 4.73±0.21 | 9.49±0.41 | 1.01±0.08 | m |
| 2 | 5.36±0.31 | 4.85±0.35 | 10.21±0.32 | 1.11±0.10 | m |
| 3 | 4.88±0.28 | 4.60±0.25 | 9.49±0.26 | 1.06±0.06 | m |
| 4 | 5.26±0.37 | 4.66±0.41 | 9.93±0.40 | 1.13±0.09 | m |
| 5 | 4.70±0.27 | 3.97±0.20 | 8.67±0.24 | 1.18±0.09 | m |
| 6 | 5.04±0.34 | 3.81±0.24 | 8.86±0.30 | 1.32±0.11 | m |
| 7 | 4.10±0.29 | 3.84±0.28 | 7.94±0.30 | 1.07±0.08 | m |
| 8 | 4.51±0.30 | 3.78±0.19 | 8.29±0.26 | 1.19±0.10 | m |
| 9 | 4.70±0.22 | 0 | 4.70±0.22 | ∞ | T |
| 10 | 4.66±0.23 | 0 | 4.66±0.23 | ∞ | T |
| 11 | 4.48±0.31 | 0 | 4.48±0.31 | ∞ | T |
| 12 | 4.73±0.28 | 0 | 4.73±0.28 | ∞ | T |
| 13 | 4.41±0.31 | 0 | 4.41±0.31 | ∞ | T |
| 14 | 4.16±0.26 | 0 | 4.16±0.26 | ∞ | T |

**LL** relative length of long arm. **SL** relative length of short arm. **TL** total relative length. **SD** standard deviation.

**Supplementary Table S3** Measurements of somatic chromosomes of natural hybrid 1

| No. | Relative length (%) | | | Arm ratio (LL/SL) ±SD | Type |
| --- | --- | --- | --- | --- | --- |
|  | LL±SD | SL±SD | TL±SD |  |  |
| 1 | 5.32±0.38 | 4.45±0.27 | 9.77±0.44 | 1.20±0.09 | m |
| 2 | 6.74±0.41 | 5.32±0.39 | 12.06±0.48 | 1.27±0.08 | m |
| 3 | 5.50±0.42 | 4.96±0.32 | 10.46±0.45 | 1.11±0.05 | m |
| 4 | 4.45±0.35 | 3.90±0.30 | 8.35±0.35 | 1.14±0.05 | m |
| 5 | 5.50±0.31 | 0 | 5.50±0.31 | ∞ | T |
| 6 | 5.12±0.28 | 0 | 5.12±0.28 | ∞ | T |
| 7 | 4.18±0.30 | 0.25±0.02 | 4.43±0.31 | 16.72±0.81 | t |
| 8 | 4.70±0.26 | 0 | 4.70±0.26 | ∞ | T |
| 9 | 4.04±0.28 | 0.61±0.03 | 4.65±0.30 | 6.62±0.38 | st |
| 10 | 3.83±0.23 | 0.53±0.02 | 4.36±0.24 | 7.23±0.34 | t |
| 11 | 3.48±0.19 | 0.79±0.04 | 4.27±0.21 | 4.41±0.29 | st |
| 12 | 3.46±0.20 | 0.74±0.06 | 4.20±0.25 | 4.68±0.31 | st |
| 13 | 3.10±0.23 | 0.53±0.03 | 3.63±0.22 | 5.85±0.42 | st |
| 14 | 3.51±0.27 | 0.50±0.02 | 4.01±0.28 | 7.02±0.32 | t |
| 15 | 3.42±0.25 | 0.39±0.02 | 3.81±0.25 | 8.77±0.21 | t |
| 16 | 3.19±0.16 | 0.48±0.03 | 3.67±0.20 | 6.65±0.35 | st |
| 17 | 2.84±0.12 | 0.71±0.04 | 3.55±0.15 | 4.00±0.23 | st |
| 18 | 3.30±0.21 | 0.16±0.01 | 3.46±0.20 | 20.63±1.26 | t |

**LL** relative length of long arm. **SL** relative length of short arm. **TL** total relative length. **SD** standard deviation.

**Supplementary Table S4** Measurements of somatic chromosomes of natural hybrid 2

| No. | Relative length (%) | | | Arm ratio (LL/SL) ±SD | Type |
| --- | --- | --- | --- | --- | --- |
|  | LL±SD | SL±SD | TL±SD |  |  |
| 1 | 7.36±0.50 | 6.09±0.48 | 13.45±0.91 | 1.21±0.01 | m |
| 2 | 5.06±0.29 | 4.59±0.28 | 9.65±0.32 | 1.10±0.01 | m |
| 3 | 5.54±0.31 | 4.79±0.31 | 10.33±0.45 | 1.16±0.08 | m |
| 4 | 4.45±0.28 | 4.43±0.27 | 8.88±0.32 | 1.01±0.01 | m |
| 5 | 5.14±0.36 | 0 | 5.14±0.36 | ∞ | T |
| 6 | 5.11±0.31 | 0 | 5.11±0.31 | ∞ | T |
| 7 | 4.67±0.28 | 0.17±0.01 | 4.84±0.28 | 27.47±2.04 | t |
| 8 | 4.83±0.32 | 0 | 4.83±0.32 | ∞ | T |
| 9 | 3.97±0.26 | 0.59±0.04 | 4.56±0.29 | 6.73±0.36 | st |
| 10 | 3.90±0.30 | 0.59±0.02 | 4.49±0.30 | 6.63±0.54 | st |
| 11 | 3.78±0.24 | 0.19±0.01 | 3.97±0.25 | 19.89±1.69 | t |
| 12 | 3.72±0.23 | 0.20±0.01 | 3.92±0.25 | 18.60±1.58 | t |
| 13 | 3.37±0.28 | 0.24±0.01 | 3.61±0.26 | 14.04±1.12 | t |
| 14 | 3.24±0.19 | 0.51±0.02 | 3.75±0.21 | 6.35±0.40 | st |
| 15 | 2.90±0.15 | 0.66±0.02 | 3.56±0.19 | 4.39±0.31 | st |
| 16 | 3.40±0.18 | 0.24±0.01 | 3.64±0.17 | 14.17±1.04 | t |
| 17 | 2.60±0.20 | 0.54±0.04 | 3.14±0.22 | 4.81±0.32 | st |
| 18 | 2.58±0.17 | 0.54±0.03 | 3.12±0.21 | 4.78±0.30 | st |

**LL** relative length of long arm. **SL** relative length of short arm. **TL** total relative length. **SD** standard deviation.

**Supplementary Table S5** The chloroplast genome SSR loci distributions of four samples in *Lycoris*

| SSR Type |  | SSR Repeat Base | | | |  | SSR Start | | | |
| --- | --- | --- | --- | --- | --- | --- | --- | --- | --- | --- |
|  | *L. aurea* | | Natural hybrid 1 | Natural hybrid 2 | *L. radiata* | *L. aurea* | | Natural hybrid 1 | Natural hybrid 2 | *L. radiata* |
| Mono-  nucleotide | (A)11 | | (A)12 | (A)11 | (A)12 | 3580 | | 3584 | 3580 | 3584 |
|  | (T)11 | | - | (T)11 | - | 3701 | | - | 3701 | - |
|  | (T)10 | | (T)10 | (T)10 | (T)10 | 4632 | | 4634 | 4632 | 4634 |
|  | (C)11 | | (C)10 | (C)11 | (C)10 | 4879 | | 4881 | 4879 | 4881 |
|  | (A)10 | | (A)10 | (A)10 | (A)10 | 7475 | | 7470 | 7475 | 7470 |
|  | (T)10 | | (T)10 | (T)10 | (T)10 | 8157 | | 8152 | 8157 | 8152 |
|  | (A)10 | | - | (A)10 | - | 8381 | | - | 8381 | - |
|  | (T)12 | | (T)13 | (T)12 | (T)13 | 10615 | | 10606 | 10615 | 10606 |
|  | - | | (A)11 | - | (A)11 | - | | 14137 | - | 14137 |
|  | (T)16 | | (T)18 | (T)16 | (T)18 | 17031 | | 17006 | 17031 | 17006 |
|  | (T)14 | | (T)13 | (T)13 | (T)13 | 23149 | | 23126 | 23149 | 23126 |
|  | (A)10 | | (A)10 | (A)10 | (A)10 | 30004 | | 29979 | 3003 | 29979 |
|  | (A)12 | | (A)11 | (A)12 | (A)10 | 30569 | | 30544 | 30568 | 30544 |
|  | (T)11 | | - | (T)11 | - | 31004 | | - | 31003 | - |
|  | (T)11 | | (T)10 | (T)11 | (T)10 | 31632 | | 31604 | 31631 | 31603 |
|  | (A)12 | | (A)13 | (A)12 | (A)13 | 33202 | | 33174 | 33201 | 33173 |
|  | - | | (A)10 | - | (A)10 | - | | 33465 | - | 33464 |
|  | (A)10 | | (A)11 | (A)10 | (A)11 | 33797 | | 33771 | 33796 | 33770 |
|  | (T)10 | | (T)11 | (T)10 | (T)11 | 34210 | | 34185 | 34209 | 34184 |
|  | (A)10 | | (A)10 | (A)10 | (A)10 | 37763 | | 37739 | 37762 | 37738 |
|  | (A)12 | | (A)12 | (A)12 | (A)12 | 46918 | | 46913 | 46917 | 46912 |
|  | - | | (T)12 | - | (T)12 | - | | 52907 | - | 52906 |
|  | (T)10 | | (T)10 | (T)10 | (T)10 | 69128 | | 69129 | 69127 | 69128 |
|  | - | | (C)10 | - | (C)10 | - | | 69886 | - | 69885 |
|  | (T)13 | | (T)14 | (T)13 | (T)14 | 70228 | | 70230 | 70227 | 70230 |
|  | (T)10 | | (T)10 | (T)10 | (T)10 | 71111 | | 71114 | 71110 | 71114 |
|  | - | | (A)10 | - | (A)10 | - | | 72920 | - | 72920 |
|  | (T)11 | | (T)12 | (T)11 | (T)12 | 73201 | | 73201 | 73200 | 73201 |
|  | (A)12 | | (A)12 | (A)12 | (A)12 | 73985 | | 73986 | 73984 | 73986 |
|  | (A)10 | | - | (A)10 | - | 74656 | | - | 74655 | - |
|  | (T)10 | | - | (T)10 | - | 76634 | | - | 76633 | - |
|  | (A)10 | | (A)10 | (A)10 | (A)10 | 80564 | | 80563 | 80563 | 80563 |
|  | (A)10 | | (A)10 | (A)10 | (A)10 | 103205 | | 103206 | 103204 | 103206 |
|  | (T)14 | | (T)14 | (T)14 | (T)14 | 115572 | | 115571 | 115571 | 115571 |
|  | (A)15 | | (A)13 | (A)15 | (A)12 | 115852 | | 115851 | 115851 | 115851 |
|  | (A)11 | | - | (A)11 | - | 115998 | | - | 115997 | - |
|  | (T)10 | | - | (T)10 | - | 116353 | | - | 116352 | - |
|  | (T)10 | | - | (T)10 | - | 122106 | | - | 122105 | - |
|  | (A)10 | | (A)10 | (A)10 | (A)10 | 130557 | | 130567 | 130564 | 130567 |
|  | (T)10 | | (T)10 | (T)10 | (T)10 | 141789 | | 141799 | 141796 | 141799 |
| di-nucleotide | (AT)5 | | (AT)5 | (AT)5 | (AT)5 | 20491 | | 20468 | 20491 | 20468 |
|  | (TA)7 | | (TA)7 | (TA)7 | (TA)7 | 37266 | | 37242 | 37265 | 37241 |
|  | (AT)5 | | (AT)5 | (AT)5 | (AT)5 | 48814 | | 48806 | 48813 | 48805 |
|  | (GA)5 | | (GA)5 | (GA)5 | (GA)5 | 92470 | | 92471 | 92469 | 92471 |
|  | (TA)5 | | (TA)5 | (TA)5 | (TA)5 | 117151 | | 117145 | 117150 | 117144 |
|  | (TC)5 | | (TC)5 | (TC)5 | (TC)5 | 152524 | | 152534 | 152531 | 152534 |
| Tri-nucleotide | (ATT)5 | | (ATT)5 | (ATT)5 | (ATT)5 | 116682 | | 116676 | 116681 | 116675 |
| Tetra-  nucleotide | (TTTA)3 | | (TTTA)3 | (TTTA)3 | (TTTA)3 | 9610 | | 9614 | 9610 | 9614 |
|  | (TTTA)3 | | (TTTA)3 | (TTTA)3 | (TTTA)3 | 32243 | | 32215 | 32242 | 32214 |
|  | (TTTC)3 | | (TTTC)3 | (TTTC)3 | (TTTC)3 | 43514 | | 43490 | 43513 | 43489 |
|  | (TTCT)3 | | (TTCT)3 | (TTCT)3 | (TTCT)3 | 44965 | | 44960 | 44964 | 44959 |
|  | (AATG)3 | | (AATG)3 | (AATG)3 | (AATG)3 | 64474 | | 64474 | 64473 | 64473 |
|  | (AATA)3 | | (AATA)3 | (AATA)3 | (AATA)3 | 118906 | | 118900 | 118905 | 118899 |
| Penta-  nucleotide | (TTTAT)3 | | - | (TTTAT)3 | - | 63574 | | - | 63573 | - |
|  | (GGAAA)3 | | (CGAAA)3 | (CGAAA)3 | (CGAAA)3 | 111168 | | 111169 | 111167 | 111169 |
|  | (TTTCG)3 | | (TTTCG)3 | (TTTCG)3 | (TTTCG)3 | 133821 | | 133831 | 133828 | 133831 |
| Compound  sequence | CS 1 | | CS 1 | CS1 | CS 1 | 13768 | | 13745 | 13768 | 13745 |
|  | CS2 | | - | CS2 | - | 14160 | | - | 14160 | - |
|  | CS3 | | CS3 | CS3 | CS3 | 18990 | | 18967 | 18990 | 18967 |
|  | CS4 | | CS5 | CS4 | CS5 | 48402 | | 48397 | 48401 | 48396 |
|  | CS6 | | CS7 | CS6 | CS7 | 57390 | | 57385 | 57389 | 57384 |
|  | CS8 | | CS8 | CS8 | CS8 | 59809 | | 59808 | 59808 | 59807 |
|  | - | | CS9 | - | CS9 | - | | 63573 | - | 63572 |
|  | CS10 | | CS10 | CS10 | CS10 | 63798 | | 63798 | 63797 | 63797 |
|  | CS11 | | CS12 | CS11 | CS12 | 68338 | | 68338 | 68337 | 68337 |
|  | CS13 | | - | CS13 | - | 72904 | | - | 72903 | - |
|  | CS14 | | CS15 | CS14 | CS16 | 120875 | | 120869 | 120874 | 120868 |
|  | CS17 | | CS17 | CS17 | CS17 | 126874 | | 126881 | 126881 | 126881 |
|  | CS18 | | CS18 | CS18 | CS18 | 128102 | | 128112 | 128109 | 128112 |

**CS1**:(TA)5ataaatatatataatataaataac(ATAA)3;**CS2**:(A)12ttctga(T)10;**CS3**: (T)10gattccttagaa(T)10

ccgttcccggtggtatcaaaatgccgctgtgtttggatatcttatctgtctccccgggaaaatgaatatctccagaaaatattttcagttcaatac(T)11; **CS4**: (T)14agttgagaattttaattattttgatatata(T)12; **CS5**: (T)11agttgagaattttaattattttg(AT)5(T)10*; **CS6**:(TA)5gt(TA)5aagtagaaagataagt(TA)5;**CS7**: (TA)5gt(TA)5aagtagaaagataagt(TA)7;**CS8**:(T)10

gtacatgacatgagagaaacctgtctttatatttcaaatttaggaaaagattccatca(AT)5;**CS9**:(TTTAT)3atttctatattttatata(T)10;**CS10**:(A)10gaaagcattgacttccctcccatatcttgcatctatagtatttttgccctggtggg(TC)5;**CS11**: (A)11tgaaaa

taaaggtttttttctacctatgtaatttgtagttcta(T)10agtgctatctatttctgtcaattgattttttcttaccgtcgtatctg(A)11;**CS12**: (A)12tgaaaataaaggtttttttctacctatgtaatttgtagttcta(T)10agtgctatctatttctgtcaattgattttttcttaccgtcgtatctg(A)11;**CS13**: (A)11gaag(A)10tagaattgaacatccgtacgggcatcttttgcgcattgcatacggctccgcaatggaatttcctttttctt

cccttctattctatatcgaagc(A)10;**CS14**: (A)12tgaaataatttaaataaaattttttatatatttttttatatatttttttatatatttttatattt

atatattataaaatttata(TATAT)3;**CS15**: (A)17tgaaataatttaaataaaattttttatatatttttttatatatttttttatatatttttatatttat

atattataaaa tttata(TATAT)3;**CS16**:(A )18tgaaataatttaaataaaattttttatatatttttttatatatttttttatatatttttatatttata

tattataaaatttata(TATAT)3;**CS17**:(TTA)4tatcagttatttccagttatttgaatctagatacacaaaaatttttgatttcttcatatacaa

tata(T)10attttatccaaatcaaattgcaaatttgatttggatagaaagggatgatacatttatgcattcagggaagagaataa(T)10;**CS18**:(T)11gttggaattttctaattcaagatata cttttttattagttaatataggaaga(T)11.
